# Postoperative Bildgebung der Wirbelsäule

**DOI:** 10.1007/s00117-022-01034-2

**Published:** 2022-07-05

**Authors:** S. Schlaeger, J. S. Kirschke

**Affiliations:** Abteilung für Diagnostische und Interventionelle Neuroradiologie, Klinikum rechts der Isar, Ismaninger Str. 22, 81675 München, Deutschland

**Keywords:** Wirbelsäulenoperation, Instrumentierung, Metallartefaktreduktion, Operationserfolg, Komplikationen, Spine surgery, Instrumentation, Metal artifact reduction, Treatment outcome, Complications

## Abstract

Die Bildgebung der postoperativen Wirbelsäule hat im Wesentlichen zwei Aufgaben: Sie dient der Kontrolle des operativen Erfolgs und der Identifikation von Komplikationen. Dafür stehen die konventionelle Röntgenaufnahme, Computertomographie (CT), Myelographie und Magnetresonanztomographie (MRT) zur Verfügung. Unter Berücksichtigung der präoperativen Situation, der durchgeführten Operation und der postoperativen Beschwerdekonstellation ist es Aufgabe der Radiologinnen und Radiologen, die passende Modalität für eine suffiziente Diagnostik zu wählen. Insbesondere der Zustand nach Implantation von Fremdmaterial bedeutet eine technische Herausforderung im Rahmen der Bildakquisition. In der Befundung sehen sich die Radiologinnen und Radiologen mit der Aufgabe konfrontiert, zwischen natürlichen, zu erwartenden postoperativen Veränderungen und relevanten Komplikationen zu differenzieren. Ein reger Austausch mit Patientinnen und Patienten und zuweisenden Klinikerinnen und Klinikern ist dabei unerlässlich. Insbesondere klinische Hinweise auf einen Infekt, neue oder deutliche progrediente neurologische Ausfallserscheinungen und das Konus-Kauda-Syndrom erfordern eine zeitnahe Diagnosestellung, um eine rasche Therapieeinleitung zu gewährleisten.

## Abstract (en)

Imaging of the postoperative spine intends to answer two main questions: It is used to assess the surgical success and to identify complications. To this end, conventional X‑ray, computer tomography (CT), myelography, and magnetic resonance imaging (MRI) are available. The radiologist has to select the appropriate modality for sufficient diagnosis considering the preoperative situation, the performed operation, and existing postoperative symptoms. Particularly, the implantation of foreign material represents a technical challenge in the context of image acquisition. In the radiologist’s report, one must differentiate between expected postoperative changes and relevant complications. Close communication with the patients and the referring clinicians is essential. Especially clinical signs of infection, new or progressive neurological deficits and cauda equina and conus medullaris syndromes require an immediate diagnosis to facilitate prompt therapy.

Wirbelsäuleneingriffe gehören zu den häufigsten Operationen, sodass die konsekutive bildgebende Beurteilung einen hohen Stellenwert hat. Neben einer fundierten Kenntnis der Befundkonstellation der Patientin bzw. des Patienten und der durchgeführten Operation spielt vor allem die Wahl der richtigen Untersuchungstechnik eine tragende Rolle. Diese Übersicht soll eine Entscheidungshilfe darstellen. In einen guten radiologischen Befund werden alle wesentlichen Aspekte aufgenommen: Zustand der anatomischen Strukturen und der ggf. eingebrachten Instrumentierung, sowie eine Beurteilung des operativen Erfolgs und die Identifikation potenzieller Komplikationen.

## Untersuchungstechniken

Neben Untersuchungstechniken, die speziellen Fragestellungen vorbehalten sind, wie Ultraschall, Single-Photon-Emissions-Computertomographie (SPECT-CT) und Positronen-Emissions-Tomographie (PET), stehen den Radiologinnen und Radiologen hauptsächlich vier Modalitäten zur Verfügung: konventionelle Röntgenuntersuchung, CT, CT-Myelographie und Magnetresonanztomographie (MRT) [4]. Die Wahl der richtigen Untersuchungstechnik hängt von der initialen Pathologie und der präoperativen Symptomatik ab, sowie vom operativen Zugangsweg und Operationsverfahren, dem Intervall zwischen Eingriff und eventuellem postoperativem Symptombeginn, sowie der Art der aktuell vorliegenden Symptomatik.

### Cave.

Bei unklaren Befunden kann neben der obligaten Korrelation zum klinischen Beschwerdebild auch die Kombination mehrerer bildgebender Modalitäten erforderlich sein.

## Röntgenuntersuchung

Die konventionelle Röntgenaufnahme, meist aufgenommen in anterior-posteriorer (a.-p.) und lateraler Projektion, wird hauptsächlich zur Kontrolle der postoperativen Implantatlage und -integrität verwendet [42]. Die breite Verfügbarkeit und die rasche Durchführung machen dieses Verfahren damit zur ersten Wahl bei postoperativen Routineuntersuchungen [8]. Ein weiterer Vorteil ist die Möglichkeit, die Wirbelsäule unter Belastung, also beim stehenden Patienten abzubilden. Aufnahmen in Extensions- und Flexionsstellung erlauben eine dynamische Stellungskontrolle der Wirbelsäulenverhältnisse, sind aber postoperativ selten indiziert [45]. Im Gegensatz zur guten Beurteilbarkeit der Implantate und Stellungsverhältnisse ist eine differenziertere Beurteilung diskreter ossärer Veränderungen wie nichtdislozierter Frakturen, der Verhältnisse im Spinalkanal, in den Neuroforamina oder in den angrenzenden Weichteilen nicht möglich [14].

Kommt es jedoch zu einer klinischen Verschlechterung, sind gerade die unmittelbare Implantatumgebung und eine genaue Befundung des Operationsgebiets von besonderem diagnostischem Interesse. Um diesen Anforderungen gerecht zu werden, eignen sich verschiedene Verfahren der Schnittbildgebung.

## Computertomographie

Der ausgezeichnete Knochenkontrast der CT gestattet eine sorgfältige Beschreibung des Wirbelsäulenalignments, der Knochenkontinuität, degenerativer und traumatischer ossärer Veränderungen, spinaler und neuroforaminaler Engen, und von Implantatposition und -intaktheit, sowie die Inkorporation in die ossären Strukturen. Auch die Weichteilstrukturen können zu einem begrenzten Maß beurteilt werden. So können größere postoperative Blut‑/Flüssigkeitsansammlungen nachgewiesen oder der Verdacht auf eine Infektion gestellt werden. Moderne Multidetektor-CT (MDCT) Geräte liefern hochaufgelöste, multiplanare und 3D-Rekonstruktionen zur dedizierten Befundung auch subtiler Veränderungen in kurzer Scanzeit [7].

### Metallartefaktreduktion

Das eingebrachte Fremdmaterial bedeutet eine Herausforderung an die Aufnahmetechnik [30]. Dabei werden als Hauptverursacher von Metallartefakten die verstärkte Absorption von Röntgenphotonen (*Photon-Starvation-Effekt*) und die Aufhärtung des Röntgenstrahlenspektrums (Aufhärtungsartefakte) unterschieden [25, 36]. Zusätzlich schränken vermehrte Streustrahlung, Partialvolumeneffekte und Artefakte an den Grenzflächen die Bildqualität ein. Metallartefaktreduktion kann zum einen durch eine Optimierung der Aufnahmeparameter bei der Bildakquisition und zum anderen durch spezielle Nachverarbeitungsmethoden und dedizierte Aufnahmetechniken erreicht werden [13]. Eine Erhöhung der Röhrenspannung und des Röhrenstroms erhöhen die Wahrscheinlichkeit, dass Röntgenstrahlen das Metallimplantat passieren, bei aber deutlich erhöhter Strahlenbelastung der Patientin bzw. des Patienten [25]. Dünnere Kollimatoren helfen bei der Reduktion von Partialvolumeneffekten und Streuartefakten [25]. Die obere Begrenzung der Hounsfield Skala kann auf 40.000 HU gesetzt werden, da die meisten Metalle eine größere Strahlenabsorption als die Standard Hounsfield Skalen Begrenzung aufweisen [27]. Bei der iterativen Rekonstruktion werden mehrfache Korrekturschleifen zur Reduktion von Bildrauschen und Artefakten durchgeführt [3]. Mittlerweile bieten die meisten Hersteller solche Artefaktkorrekturen an [11]. Aufhärtungsartefakte lassen sich zumindest partiell mittels moderner Dual-Energy-CTs reduzieren: Hier werden zwei Datensätze mit unterschiedlichem polychromatischem Energiespektrum akquiriert, woraus ein virtuelles Bild mit monochromatischem Energiespektrum berechnet wird, was zu einer deutlichen Reduktion der Aufhärtungsartefakte führt [12].

### CT-Myelographie

**Figure 1:**
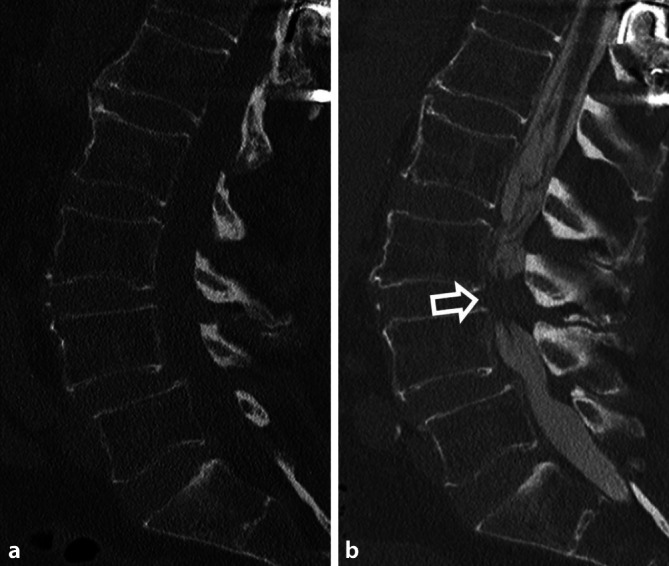

Bei der CT-Myelographie wird die CT mit einer intrathekalen Gabe von Kontrastmittel kombiniert [26]. Die Verteilung des Kontrastmittels im spinalen Liquorraum gestattet die Beurteilung des Myleons, der Kaudafasern und der Wurzeltaschen in Kombination mit einer durch den CT-Kontrast gegebenen Beurteilung der knöchernen Strukturen. So können z. B. eine Kompression des Myelons oder der Nervenwurzeln bei diskoligamentären Insuffizienzen detektiert werden (Abb. 1). Die Technik kommt insbesondere dann zur Anwendung, wenn eine MRT aufgrund von Metallartefakten nicht verwertbar ist oder MRT-Kontraindikationen, wie z. B. ein Herzschrittmacher bestehen.

Die Notwendigkeit des Einsatzes von ionisierender Strahlung und eine erschwerte Beurteilung des Weichteilgewebes bleiben jedoch Nachteile der CT.

## Magnetresonanztomographie

### Cave.

Der exzellente Weichteilkontrast, die Möglichkeit zur multiparametrischen Darstellung und die fehlende Strahlenbelastung machen die MRT zum Goldstandard der Wirbelsäulenbildgebung [4].

Mit dieser Technik können neben Myelon, Nervenwurzeln und paravertebralem Weichteilgewebe auch knöcherne Strukturen durch den Nachweis von Knochenmarködem beurteilt werden [4]. So lassen sich einige Pathologien (z. B. Syringomyelien) nur mittels MRT darstellen und insbesondere klinisch akut relevante Komplikationen, wie Blutungen oder Infektionen, können sicher diagnostiziert werden [17, 23, 26]. Wirbelsäulenuntersuchungsprotokolle beinhalten je nach Fragestellung sagittale T2-, fettgesättigte T2- und T1-Sequenzen mit/ohne Kontrastmittel, gefolgt von einer axialen T2-/T1-Bildgebung relevanter Befunde und ggf. ergänzt durch eine koronare Darstellung, was insbesondere die Befundung der paravertebralen Strukturen erleichtert.

### Metallartefaktreduktion

Auch in der MRT stören Implantate oder Schraubenabrieb die Bildqualität durch sog. Suszeptibilitätsartefakte [39]. Da für eine gute Bildqualität ein homogenes Magnetfeld essenziell ist, führen Fremdmaterialen, die das lokale Magnetfeld stören, zu Signalverzerrung/-verschiebung, Signalverlust oder fokaler Signalverstärkung [15]. Diese Artefakte können dabei sowohl aus der Ebene selbst („in plane“) oder aus einer benachbarten Ebene („through plane“) stammen. Neben der Verwendung von neuartigen Instrumentierungsmaterialien wie PEEK (Polyetheretherketone), einem Polymer, das sehr wenige Artefakte in der MRT verursacht, können die Standardsequenzen optimiert werden. Aufgrund des direkt proportionalen Zusammenhangs zwischen Magnetfeldstärke und Artefaktausmaß gilt allgemein, dass die 1,5 T Bildgebung der 3 T Bildgebung bei Metallimplantaten überlegen ist. Auch sind schnelle Spin-Echo-Sequenzen besser geeignet als Gradienten-Echo-Sequenzen, da die multiplen 180° Refokussierungspulse von z. B. Turbo-Spin-Echo-Sequenzen zu einer Verringerung der durch die Feldverzerrung verursachten Dephasierung der Spins führen. Weitere einfache Methoden der Artefaktkorrektur sind die Erhöhung der Empfängerbandbreite und der Bandbreite des Anregungspulses, die Wahl dünnerer Schichten und kleinerer Voxelgrößen sowie die Verwendung von STIR(„short-tau inversion recovery“)-Fettunterdrückung (basierend auf einem optimierten Inversionspuls) oder Dixon-Fettseparation im Gegensatz zu frequenzselektiver Fettsättigung [15, 37, 43]. Zu den fortgeschrittenen Methoden der Metallartefaktreduktion gehört das View-Angle-Tilting (VAT) [15]. Der zusätzliche Gradient kompensiert die durch das Metall induzierte Verzerrung. Ebenenübergreifende Artefaktkorrekturen sind Techniken wie „slice encoding for metal artifact correction“ (SEMAC) und „multi-acquisition variable-resonance image combination“ (MAVRIC). Bei SEMAC erlaubt die zusätzliche Phasenkodierung, entlang der Schichtselektion eine Verzerrung der angrenzenden Schichten zu reduzieren [29]. Kombiniert man SEMAC mit einer zusätzlichen Erhöhung der Empfängerbandbreite und dem Turbo-Spin-Echo der VAT-Technik, spricht man von WARP, was auch eine Korrektur der Artefakte innerhalb der Bildebene erlaubt. Bei MAVRIC werden 3D-Messungen mit überlappenden Frequenzen akquiriert, welche anschließend zusammengerechnet werden, um das artefaktreduzierte Bild zu erzeugen [24]. Nachteile dieser Techniken sind die deutlich verlängerten Aufnahmezeiten. Auch können zwar Verzerrungen reduziert, aber Signalauslöschungen nicht kompensiert werden, wie sie oft zwischen Stäben, Schrauben und Wirbelkörperersatz entstehen. In solchen Fällen hilft oft nur die CT-Myelographie weiter.

Eine Übersicht der Vor- und Nachteile der vier Hauptmodalitäten zur Beurteilung der postoperativen Wirbelsäule ist in Tab. 1 dargestellt.

**Table 1:** 

	Vorteile	Nachteile
Konventionelle Röntgenaufnahme	– Breite Verfügbarkeit – Stellungskontrolle – Lagekontrolle und Intaktheit der Instrumentierung – Materiallockerung – Dynamische Beurteilung: Flexion, Extension etc.	– Keine 3D-Darstellung – Projektionstechnik – Limitierte Darstellung der Weichteilstrukturen
CT	– Breite Verfügbarkeit – Multiplanare und 3D-Rekonstruktionen – Exzellenter Knochenkontrast – Bewertung der Instrumentierung	– Metallartefakte – Schwierig akute von chronischen Veränderungen zu unterscheiden – Strahlenbelastung
CT-Myelographie	– Dynamische Beurteilung – Beurteilung von Myelon, Kaudafasern und Wurzeltaschen sowie von knöchernen Strukturen	– Invasive, intrathekale Kontrastmittelgabe
MRT	– Exzellenter Weichteilkontrast – Darstellung von Entzündung und Flüssigkeitsansammlungen (Komplikationen!) – Keine Strahlenbelastung	– Metallartefakte – Kontraindikationen, wie z. B. Schrittmacher – Nur indirekte Beurteilung knöcherner Strukturen (Knochenmarködem)

## Komplikationen

Neben der Beurteilung des operativen Erfolgs (Stellungskontrolle, Implantatlage, Dekompressionsstatus, Resektionsstatus) ist vor allem die zeitnahe Identifikation und richtige Einordnung von akuten oder chronischen postoperativen Komplikationen die Hauptaufgabe der Radiologinnen und Radiologen. Dabei treten Infektionen und neurologische Komplikationen vorwiegend in den ersten drei postoperativen Monaten auf, Implantatversagen ab dem zweiten postoperativen Jahr [6]. Insbesondere die Abgrenzung früher postoperativer Komplikationen von natürlicherweise zu erwartenden postoperativen Veränderungen kann eine Herausforderung bedeuten. Dabei erlaubt die Gesamtkonstellation aus Symptomen und deren zeitlicher Entwicklung, Ergebnis klinischer Untersuchung und Laborparametern, sowie Intervall zwischen Operation und Beschwerdebeginn, im Kontext der präoperativen Situation, bereits einen ersten Eindruck im Hinblick auf die Dringlichkeit.

### Cave.

Absolute *Red Flags* sind klinische Hinweis auf einen Infekt, neue oder deutlich progrediente akute neurologische Ausfallssymptome, sowie das Konus-Kauda-Syndrom mit Blasen‑/Mastdarmstörung, welche eine sofortige Abklärung notwendig machen.

**Figure 2:**
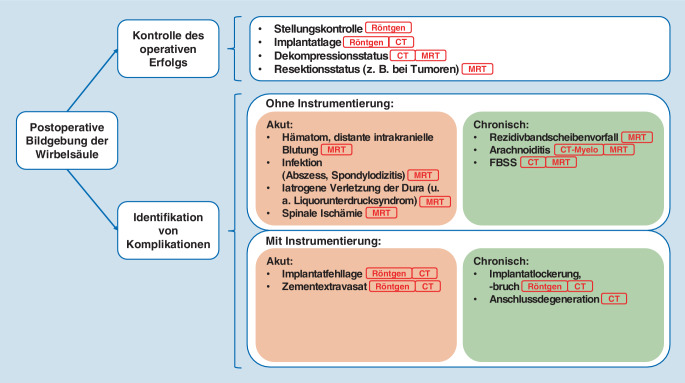

In Abb. 2 wird ein Überblick über die häufigsten bildmorphologischen, postoperativen Komplikationen und die jeweils am besten geeigneten Untersuchungsmodalitäten gegeben.

### Hämatom

Dezente Blutansammlungen im operativen Zugangsweg finden sich in fast allen postoperativen Bildern. Klinisch relevante, große und progrediente Hämatome treten in 0–1 % der Fälle auf [10]. Dabei sind epidurale Blutungen mit neuer neurologischer Symptomatik mit einer hohen Morbidität verbunden und erfordern, um die dauerhafte Schädigung zu begrenzen, eine schnelle Diagnosestellung und notfallmäßige Revision.

**Figure 3:**
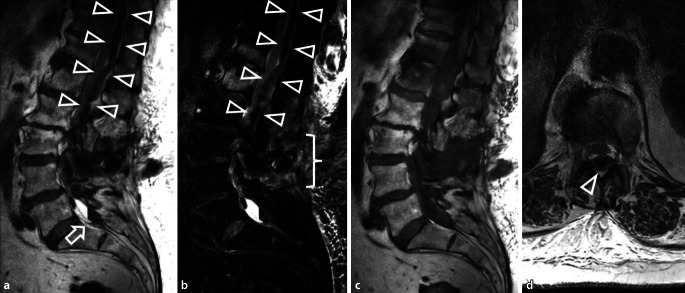

Als Faustregel gilt, dass sich Hämatome mit einer Ausdehnung von weniger als einem Wirbelsäulensegment langsam spontan resorbieren, während größere, v. a. epidurale Hämatome umgehend entlastet werden sollten [17]. Dabei korreliert nicht zwangsläufig das Ausmaß der Symptomatik mit der Größe des Hämatoms. Die MRT gilt als Methode der Wahl. Eine Analyse sagittaler und axialer Bilder ermöglicht die genaue Lokalisation und eine Beurteilung des raumfordernden Effekts auf neuronale Strukturen (Abb. 3). Das T1-/T2-Signalverhalten ist abhängig vom Alter der Blutung. Hämatome zeigen ein variables Kontastmittelverhalten [32].

### Entfernte intrakranielle Blutung

Bei postoperativen Kopfschmerzen mit fokalen, neuen neurologischen Ausfällen wie Schwindel sollte man auch an entfernte intrakranielle Blutungen denken, die sehr selten nach Wirbelsäulenoperationen mit Eröffnung der Dura und intrakranieller Hypotension auftreten können [22]. In allen bislang beschriebenen Fällen lag eine Duraverletzung vor, auch wenn diese teils okkult war [1].

### Infektion (Abszess, Spondylodiszitis)

**Figure 4:**
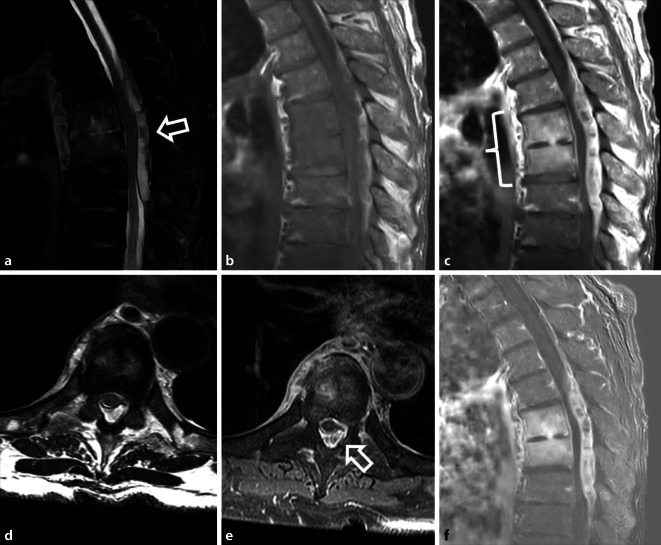

Hauptrisikofaktor für postoperative Infektionen sind höheres Alter, männliches Geschlecht, Steroidtherapie, Diabetes, Alkohol, Rauchen, Fettleibigkeit sowie perioperative Faktoren, wie hoher ASA-Score, lange Operatoinsdauer, posteriorer Zugangsweg, Operation über mehrere Segmente, hoher Blutverlust, Einsatz von Fremdmaterial und postoperative Inkontinenz [21]. Relevant ist die Unterscheidung zwischen oberflächlichen, auf die Dermis und das subkutane Gewebe begrenzten von tief reichenden, subfaszialen Infektionen. Bei Letzteren sind die Hauptpathologien paravertebrale oder epidurale Abszesse und Spondylodiszitiden mit potenzieller Ausdehnung von begleitenden paravertebralen Abszessen in die Psoasmuskulatur im Sinne von *Senkungsabzessen* oder Infektionen des Implantatlagers. Vor allem oberflächliche Infektionen zeichnen sich durch die Kardinalzeichen der Entzündung (Rubor, Calor, Tumor, Dolor und Functio laesa) aus. Insbesondere Fieber und erhöhte Entzündungsparameter (allen voran das CRP mit Erhöhung länger als zwei Wochen postoperativ und Leukozytose) sollten aufhorchen lassen und eine bildgebende Diagnostik veranlassen. Dabei bietet die MRT mit Kontrastmittelgabe die größte diagnostische Sicherheit. Abszesse stellen sich als abgekapselte Flüssigkeitskollektionen mit randständiger Kontrastmittelanreicherung und Umgebungsödem (T2-hyperintens, T1-hypointens) dar [2]. Suspekt für eine Spondylodiszitis sind eine T2-Hyperintensität, T1-Hypointensität und Kontrastmittelanreicherung der affektierten Bandscheibe und der angrenzenden, häufig erosiv veränderten Wirbelkörper (Abb. 4) [44].

#### Cave.

Nicht zu vergessen ist die Evaluation der angrenzenden paravertebralen Muskulatur, um eventuelle Abszedierungen nicht zu übersehen (Psoasabzess).

Auch kleinere Imbibierungen des paravertebralen Fettgewebes sind sehr spezifisch und auch in der CT als Obliteration der Fettschichten zwischen Wirbelkörper und M. psoas gut zu identifizieren [9]. Die größte Schwierigkeit bleibt die Unterscheidung zwischen unspezifischen, postoperativen, reparativen Veränderungen und Seromen von relevanten, entzündlichen Veränderungen. Randständige Kontrastmittelaufnahmen um liquide Formationen und epidurale Kontrastmittelaufnahme in vom Operationsgebiet entfernten Wirbelsäulensegmenten sind jedoch hochsuspekt auf einen entzündlichen Prozess. Bei hinreichendem Verdacht auf Abszess oder Spondylodiszitis kann eine perkutane, CT-gesteuerte Punktion mit eventueller Drainageeinlage erfolgen. Meist liegt eine Infektion mit *Staphylococcus aureus, Staphylococcus epidermidis* oder *Enterococcus faecalis* vor [31].

### Iatrogene Verletzung der Dura

#### Pseudomeningozele, Liquorunterdrucksyndrom, spinale Adhäsionen und Myelonherniation

Im Rahmen von spinalen Eingriffen kann es zu einer Verletzung der Dura kommen. Ein dadurch begünstigter Austritt von Liquor aus dem Duralsack kann zum sog. Liquorunterdrucksyndrom führen. Betroffenen Patienten zeigen lageabhängige, meist biokzipital drückende bis ziehende Kopfschmerzen, welche sich in aufrechter Position verschlimmern. Zudem können Schwindel, Übelkeit und horizontale Doppelbilder durch eine Abduzensparese auftreten (Abb. 4).

**Figure 5:**
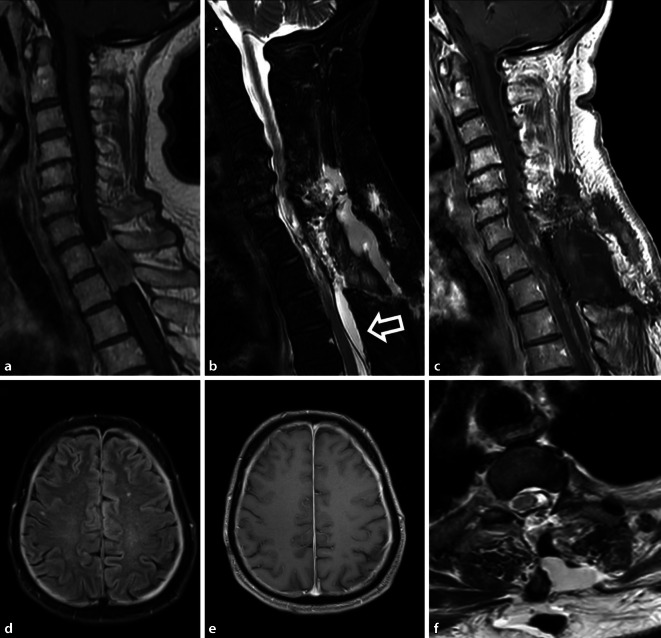

Richtungsweisend für einen spinalen Liquorverlust ist oft auch die kranielle MRT. Es zeigen sich ein meningeales Kontrastmittel-Enhancement und bei ausgeprägtem Liquorunterdruck intrakranielle Hygrome (Abb. 5; [39]). Kommt es im Operations- oder Zugangsgebiet zu einer extraduralen Kollektion von Liquor innerhalb einer Pseudokapsel, liegt eine Pseudomeningozele vor, die palpabel sein kann [38]. Typischerweise zeigt sich eine liquorisointense, zystische Formation mit Verbindung zum Duralsack. Die Abgrenzung gegenüber einem postoperativen Serom kann schwierig sein. Meist kommt es beim Liquorunterdrucksyndrom oder Pseudomeningozelen zu einer spontanen Befundregredienz innerhalb von drei Monaten. Äußerst selten sind iatrogene Herniationen des Myelons nach Eröffnung oder Verletzung der Dura zu beobachten [34]. Diese können mit einer sichtbaren Myelomeningozele einhergehen oder sich als Adhäsion des Myelons an der Dura manifestieren.

### Spinale Ischämie

**Figure 6:**
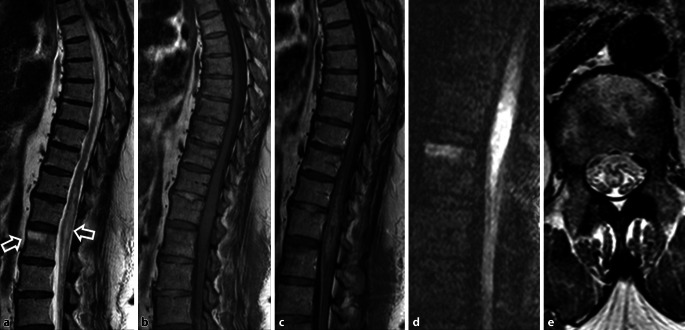

Insbesondere anteriore Operationszugangswege bergen die Gefahr der Verletzung von Segmentarterien, was zu einem Myeloninfarkt führen kann. Dabei hat vor allem der Verschluss der Adamkiewicz-Arterie, der größten zufließenden Arterie zum Rückenmark mit Versorgung des thorakolumbalen Übergangs, schwere Folgen. In der postoperativen MRT sollten ein neu aufgetretenes Wirbelkörper- oder Myelonödem in der fettgesättigten T2-gewichteten Aufnahme ggf. eine dedizierte Untersuchung mittels Diffusionsbildgebung (TSE-DWI) veranlassen (Abb. 6).

### Implantatfehllage und Zementextravasat

Um eine Implantatfehllage bei noch eröffnetem Operationssitus zu detektieren, wird die Position meist intraoperativ mittels C‑Bogen (Durchleuchtung) oder CT beurteilt, dokumentiert und kann ggf. unmittelbar korrigiert werden. Wird im Rahmen von Instrumentierungen oder Vertebro‑/Kyphoplastien Knochenzement in den betroffenen Wirbelkörper appliziert, kann es zum Austritt des Zements in intra- und paraspinale Venenplexus kommen [26]. Dies kann u. a. zu Zementembolien in der Lunge führen [18]. Schon ein postoperatives Röntgenbild erlaubt die Darstellung extrakorporaler Zementansammlungen, Embolien sind einfach in einer Lungen-CT zu detektieren. Ebenso sollten andere Fremdkörper, wie abgebrochene Bohrer oder vergessene Tupfer im postoperativen Röntgen beschrieben werden.

### Rezidiv eines Bandscheibenvorfalls

Ein erneuter Bandscheibenvorfall an gleicher Stelle kann zeitnah, aber auch erst Jahre nach erfolgter Operation auftreten. Prädilektionsstelle ist die lumbale Wirbelsäule [19]. Die Patientinnen und Patienten zeigen häufig Beschwerden, die den initialen Symptomen ähneln. Die MRT kann sowohl dabei helfen, die Größe und Lokalisation des Bandscheibenvorfalls zu beurteilen, als auch zwischen Redizivbandscheibenvorfall und narbigen postoperativen Veränderungen zu differenzieren. Die Unterscheidung gelingt meist gut mit Kontrastmittelgabe.

Narbengewebe zeigt im Unterschied zu bradytrophem, avaskulärem Bandscheibengewebe ein kräftiges Kontrastmittel-Enhancement, was jedoch im Verlauf der Jahre nach der Operation abnimmt.

### Arachnoiditis

**Figure 7:**
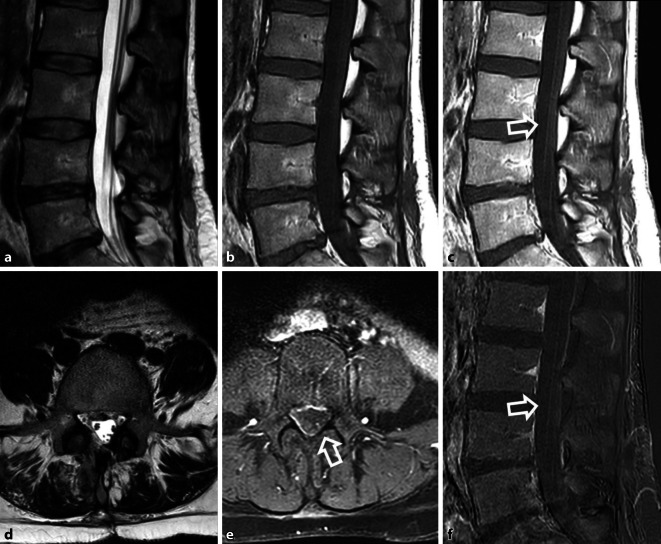

Nicht nur im Rahmen von spinalen Eingriffen, sondern auch nach entzündlichen Erkrankungen der Wirbelsäule oder intrathekaler Applikation von Medikamenten, kann es zu einer Arachnoiditis kommen [33]. Diese Entzündung der Arachnoidea zeichnet sich durch Schwellung der Nervenwurzeln mit Kollagenablagerungen und Nervenwurzelverklebung aus und kann zu stärksten, therapieresistenten Schmerzen führen. Eine CT-Myelographie hilft bei der Detektion eines durch Verklebungen behinderten Liquorflusses. Indirekte Zeichen durch Kontrastmittelaufnahme der Leptomeningen können in der MRT dargestellt werden (Abb. 7).

### Failed-Back-Surgery-Syndrom

Der Begriff *Failed-Back-Surgery-Syndrom* (FBSS) fasst unklare, persistierende Schmerzen im Rücken und Beinbereich mit eingeschränkter Funktionalität nach Wirbelsäuleneingriffen zusammen. Dem Syndrom liegen unterschiedliche Entitäten zugrunde. Wegen der multifaktoriellen Genese ist häufig eine multimodale Bildgebung mindestens mittels CT und MRT notwendig. Derzeit werden als häufigste zugrundeliegende Pathologien neuroforaminäre Stenosen, schmerzhafte Bandscheiben, Rezidivbandscheibenvorfälle, Facettengelenkarthrosen, Pseudoarthrosen, Instabilitäten und neuropathische Schmerzen diskutiert. Die Radiologin bzw. der Radiologe sollte ggf. behandelbare Ursachen identifizieren. Meist erfolgt ein multimodales Therapiekonzept, bei dem der Schmerz- und Physiotherapie ein hoher Stellenwert zukommt [5].

### Implantatlockerung, Implantatbruch

Die starke mechanische Belastung des implantierten Fremdmaterials zur Stabilisierung der Wirbelsäule kann im Verlauf zu einer Materialermüdung führen. Implantatversagen kann Ausdruck dieser Überbeanspruchung sein. Resorption von Knochengewebe kann zu schmerzhafter Schraubenlockerung oder -bruch mit korrespondierender Instabilität im betroffenen Wirbelsäulensegment führen [46]. Eine solche Lockerung kann durch Infekte hervorgerufen werden, tritt aber insbesondere bei Patienten mit Osteoporose auf [28, 40].

#### Cave.

Sowohl Röntgenbild als auch CT sind gut geeignet, um ein Implantatversagen zu detektieren.

Eine Lockerung ist gekennzeichnet durch einen Saum zwischen Implantatoberfläche und Knochenstrukturen (> 2 mm), bei Bruch zeigt sich eine typische Aufhellungslinie und ggf. Dislokation der Implantatelemente. Der Knochenkontrast der CT ist zudem gut geeignet, um eine Mitbeteiligung knöcherner Strukturen (z. B. Frakturen) zu erkennen.

### Anschlussdegeneration

**Figure 8:**
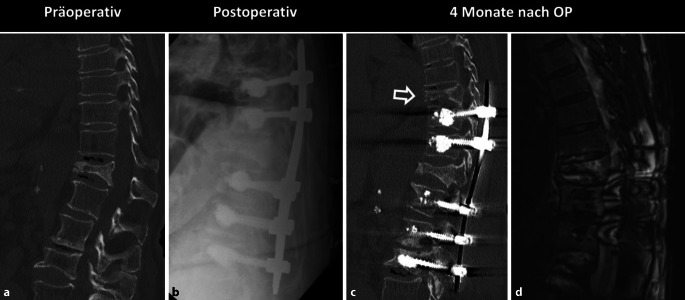

Die Veränderung der biomechanischen Eigenschaften von fusionierten Wirbelsäulensegmenten mittels Instrumentierung führt zu einer erhöhten mechanischen Belastung der angrenzenden Wirbelsäulensegmente. Dies kann zu einer sekundären Degeneration der betroffenen Wirbelkörper, Bandscheiben und Facettengelenke führen [35], bis hin zu osteoporotischen Frakturen der angrenzenden Wirbelkörper [41]. Entsprechende degenerative Veränderungen wie Malalignment, osteochondrotische Endplattenveränderungen, höhengeminderte Bandscheibenfächer, Vakuumphänomen, Bandscheibenprotrusionen/-extrusionen, Facettengelenksarthrose und Anschlussfrakturen (Abb. 8) können zuverlässig mittels CT diagnostiziert werden.

## Ausblick

Aktuell noch in der präklinischen Entwicklung stehende Techniken werden die Wirbelsäulenbildgebung in Zukunft nachhaltig beeinflussen. Dazu zählen u. a. moderne, auf künstlicher Intelligenz (KI) basierende MRT-Rekonstruktionsalgorithmen sowie die neuartige synthetische Generierung von Bildgebungskontrasten mittels Generative Adversarial Networks (GAN) [16].

## Fazit für die Praxis

- Ein radiologischer Befund zur postoperativen Wirbelsäule sollte folgende wesentliche Aspekte berücksichtigen: Vergleich der operierten Wirbelsäulensegmente mit dem präoperativen Zustand, Beurteilung normaler postoperativer Veränderungen, Erkennung von Früh- und Spätkomplikationen, ggf. Bewertung des implantierten Fremdmaterials.
- Häufig ist eine multimodale Bildgebung hilfreich.
- Techniken der Metallartefaktreduktion in Computertomographie (CT) und Magnetresonanztomographie (MRT) spielen eine tragende Rolle.
- Die Vielzahl von möglichen Interventionen und Bildgebungstechniken macht ein standardisiertes Vorgehen schwierig.
- Für Radiologinnen und Radiologen sind für die Befundung Kenntnisse von prä- und postoperativer Symptomatik, von klinischen und laborchemischen Befunden, aber auch von Operationsart und -zugangsweg, von verwendetem Material und von möglichen intraoperativen Besonderheiten essenziell.
